# Exploring the Barriers and Facilitators to Implementing a Smartphone App for Physicians to Improve the Management of Acute Myocardial Infarctions: Multicenter, Mixed Methods, Observational Study

**DOI:** 10.2196/60173

**Published:** 2025-07-08

**Authors:** Katelyn J Cullen, Hassan Mir, Madhu K Natarajan, Marija Corovic, Karen Mosleh, Jacob Crawshaw, Mathew Mercuri, Hassan Masoom, JD Schwalm

**Affiliations:** 1 Department of Medicine, McMaster University, Hamilton, ON, Canada; 2 Hamilton Health Sciences Knowledge Centre, Hamilton, ON, Canada; 3 Division of Cardiology, Ottawa Heart Institute Research Corporation, University of Ottawa Heart Institute, 40 Ruskin St, Ottawa, ON, K1Y 4W7, Canada, 1 613-696-7406; 4 Faculty of Medicine, University of Ottawa, Ottawa, ON, Canada; 5 Population Health Research Institute, Hamilton, ON, Canada; 6 Centre for Implementation Research, Ottawa Hospital Research Institute, The Ottawa Hospital, Ottawa, ON, Canada; 7 Dalla Lana School of Public Health, University of Toronto, Toronto, ON, Canada; 8 Centre for Philosophy of Epidemiology, Medicine and Public Health, Department of Philosophy, University of Johannesburg, Auckland Park, South Africa; 9 Department of Philosophy, McMaster University, Hamilton, ON, Canada; 10 Division of Cardiology, William Osler Health System, Brampton, ON, Canada

**Keywords:** ST-elevation myocardial infarction, digital health, mHealth, barriers and enablers, myocardial infarctions, myocardial, decision-making, decision, decision support, Ontario, Canada, survey, paramedics, privacy, app, application, cardiology, interprofessional communication, intervention, emergency medicine, ECG

## Abstract

**Background:**

Timely and appropriate care is critical for patients with ST-elevation myocardial infarction (STEMI). Effective communication and prompt sharing of test results, particularly electrocardiograms (ECGs), between the referring emergency medicine (EM) physician or emergency medical service (EMS) paramedic and the interventional cardiologist (IC) are essential. This exchange relies on fax or SMS text messages. The SmartAMI-ACS (Strategic Management of Acute Reperfusion and Therapies in Acute Myocardial Infarction) App was developed to streamline communication. It is user friendly and privacy compliant, and enables rapid, secure ECG sharing to support faster, informed clinical decision-making.

**Objective:**

This paper details the results of targeted preimplementation surveys to establish barriers and enablers of using a smartphone app to transmit ECG images among ICs, EM physicians, and EMS paramedics to help tailor implementation interventions.

**Methods:**

To assess the proposed acceptability and uptake of the app, preimplementation surveys were disseminated to ICs, EM physicians, and EMS paramedics in one region of Ontario, Canada. Questions were generated based on selected components of the Consolidated Framework for Implementation Research, results from a pilot study carried out at a regional hospital where the SmartAMI-ACS app was previously implemented, and predicted barriers based on expert guidance. The preimplementation surveys consisted of 7-point Likert scale questions (1=strongly disagree and 7=strongly agree) and open-ended questions. Open-ended data were extracted verbatim and analyzed using an inductive qualitative approach, with transcripts coded into descriptive qualitative codes and then collapsed into themes.

**Results:**

Survey uptake was acceptable, with 9 of the invited 10 ICs, 51 of the invited 223 EM physicians, and 93 of the invited 1138 EMS paramedics responding. All groups recognized that current practices for sharing ECGs allowed room for improvement, accepting that fax can be inconvenient and SMS text messages may not be secure. When asked whether there was a need for a smartphone app to transmit ECGs, ICs (mean 6.67, SD 0.5), EM physicians (mean 5.57, SD 1.3), and EMS paramedics (mean 5.79, SD 1.45) consistently agreed. Commonly reported barriers were concerns over technological challenges, privacy issues, and cell phone reception strength. Through the identification of the barriers in each stakeholder group, implementation strategies were developed that facilitated the scale-up of this system-change intervention.

**Conclusions:**

Results from the 3 web-based preimplementation surveys to identify key barriers and enablers to the implementation of the app helped inform the selection of tailored implementation strategies to support the rollout of the app across the health region. The surveys identified key barriers around technology, privacy, and access to required Wi-Fi that needed to be addressed during app implementation to facilitate uptake and use. Results from the surveys, and ongoing evaluation of effectiveness, are informing the expansion of the app intervention to local ambulance services and other health regions.

## Introduction

Each year, approximately 7000 people in Ontario experience ST-elevation myocardial infarctions (STEMIs) requiring timely reperfusion of the affected area of the heart to restore blood flow through the coronary arteries [1]. Reperfusion can occur by a procedure called percutaneous coronary intervention (PCI) or with thrombolytic medications. When possible, PCI is preferable, and guidelines recommend that a patient presenting to a center that can perform PCI (PCI-capable center) should have this procedure performed within 90 minutes of the patient presenting to the hospital. Those who present to a non–PCI-capable center should be transferred to a center that can perform this procedure within 120 minutes [2]. However, among the 270 health care institutions in Ontario, only 20 centers have PCI capabilities on site [3]. Thus, achieving timely care can be challenging, particularly for patients who live in rural areas where access to a PCI-capable center is limited. Where on-site PCI is not available, patients must be transferred to a partner PCI-capable hospital. Patients receiving care above evidence-based best practice time thresholds show an increased risk of mortality, repeat myocardial infarction, congestive heart failure, and hospitalization [4-7].

STEMI is diagnosed based on an electrocardiogram (ECG), and care for patients with STEMI requires rapid coordination of several services, including emergency medical services (EMS), emergency medicine (EM) physicians and staff, interventional cardiologists (ICs), and the PCI laboratory staff. This can range across several centers and jurisdictions that rarely share electronic medical records. Previous research shows that efficient coordination of these services through timely communication leads to improved outcomes for patients with STEMI [2]. The current process for STEMI activation for many centers in Canada relies on a variety of technologies that are poorly integrated or inefficient. For example, STEMI activation at the Hamilton General Hospital, a high-volume quaternary cardiac care hospital in Ontario, Canada, used fax for ECG transmission, which is secure but slow, inconvenient, and sometimes unavailable. To improve communication and efficiency in this pathway, a quick, easy-to-use, privacy-compliant smartphone app (SmartAMI-ACS [Strategic Management of Acute Reperfusion and Therapies in Acute Myocardial Infarction]) was developed for rapid ECG sharing between health care providers, enabling timely decision-making.

The SmartAMI-ACS app has been pilot tested with ICs and select EM physicians since May 2020 for patients presenting to Niagara Health and requiring transfer to Hamilton General Hospital for primary PCI. Although preliminary data show promise [8], additional research is required to promote the technology more widely as an evidence-based innovation for care. Similar smartphone apps with capabilities of sending ECG images to ICs for decision-making have demonstrated outcome improvement in patients with STEMI and a reduction in decision time [9-13]. However, there is limited evidence on users’ perspectives regarding the barriers and enablers to implementing an app that allows for the sharing of ECG images in the care of patients with STEMI, which may influence its acceptability and long-term use. To promote the app as the standard means to communicate ECGs in the setting of patients with STEMI beyond the pilot sites in the Niagara region, we sought to gather an understanding of the perceived usefulness and acceptability of the technology in routine practice from prospective app users across a wider region (ie, Southeastern Ontario) [14]. This paper details the findings from the preimplementation surveys, which will be used to assist with the potential scale-up and implementation of the app across other provincial STEMI systems in Ontario and beyond.

## Methods

### Study Design

**Figure 1.**
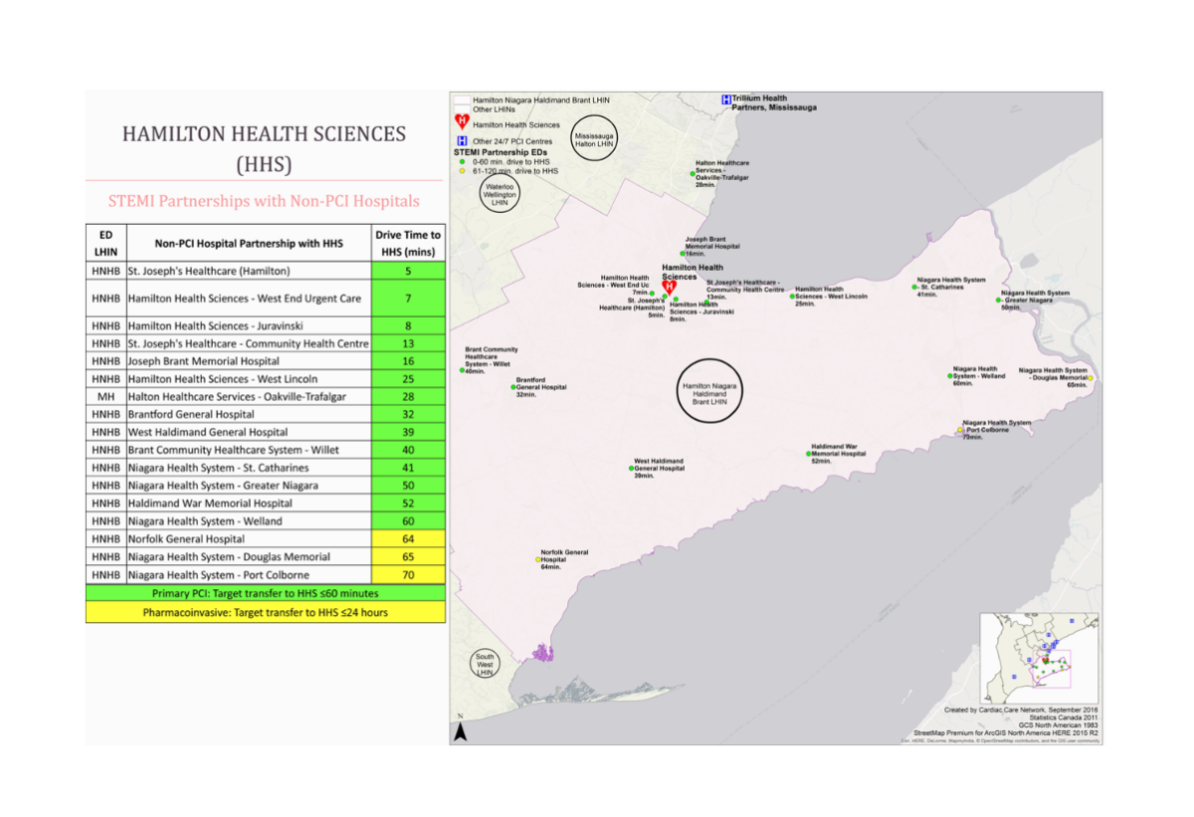
Primary PCI hospital STEMI partnership map, adapted from the 2017‐2018 Ontario Local Health Integration Network Annual Report [17]. PCI: percutaneous coronary intervention; STEMI: ST-elevation myocardial infarction.

The development and administration of the survey were led by a collaborative team consisting of medical professionals, public health researchers, and experts in implementation science. A multicenter cross-sectional study was conducted to assess the need, usefulness, and acceptability of the SmartAMI-ACS app in practice through the development and distribution of web-based surveys to key stakeholders, including ICs, EM physicians, and EMS paramedics who care for patients presenting with STEMI within the Hamilton General Hospital cardiac catheterization laboratory catchment area. The catchment area includes the Hamilton, Niagara, Haldimand, and Brant regions, which cover an area of 6473 km^2^ and a population of 1.8 million people [15,16]. Figure 1 illustrates the partner hospitals from which EM physicians were sent survey invitations, as well as a geographical map depicting the locations where sampled EMS paramedics respond to emergency calls. ICs were sampled from the primary PCI hospital indicated on the map as Hamilton Health Sciences.

### Survey Development

In 2021, three surveys were developed and tailored for each stakeholder group (ICs, EM physicians, and EMS paramedics). These surveys were focused on gathering a better understanding of the barriers and facilitators that may impact the successful implementation of the SmartAMI-ACS app intervention on a larger scale (see Multimedia Appendices 1-3 for the surveys). Development of survey questions, analysis, and interpretation were guided by results from the Niagara Health pilot study, predicted barriers based on expert guidance from study team members (including implementation science researchers, ICs, EMS paramedics, and EM physicians) and the Consolidated Framework for Implementation Research (CFIR) [18]. The CFIR is a determinant framework, with a list of 39 constructs organized across 5 major domains, and provides a practical structure to identify multilevel factors that can impact implementation [19]. CFIR is a widely used framework in implementation science, with great breadth in its application across study design, objectives, settings, and outcome measures [20]. Through an iterative process involving input from research team members, questions were drafted and selected for inclusion in the surveys around the potential need for an app to transmit ECG images, the fit of an app into existing workflow processes, the potential for an app to improve patient care and reduce treatment times, and the overall comfort level of using an app in the treatment of suspected STEMI cases. The preimplementation surveys consisted of both 7-point Likert scale questions and open-ended questions. Likert scales were used to identify participant opinions on app implementation and usefulness. We applied a 7-point scale (1=strongly disagree and 7=strongly agree), as this has been shown to reach the upper limits of the scale’s reliability, in comparison to a 5-point scale [21]. The surveys developed for each group are fully presented in Multimedia Appendices 1-3. Additional questions on the usage of the STEMI activation system (known locally as the Heart Investigation Unit Hotline) were included; however, results from those questions will not be reported in this paper.

### Sample Population

Our study targets the health care providers who use the STEMI activation system for a quaternary care hospital to activate and respond to a STEMI. These include ICs, EM physicians, and EMS paramedics. All IC and EM physicians working at one of the referring centers (Figure 1).

### Survey Distribution

The preimplementation surveys were hosted in REDCap (Research Electronic Data Capture; Vanderbilt University), and questionnaires were designed for anonymous completion. Senior leaders of each survey group used a list of physician and EMS paramedic contact details provided by administrative distribution lists to develop the survey sampling frame. Senior leaders were asked to distribute a survey invitation by email to their staff, which included a summary of the evaluation project, a link to the survey, and details about consent; they were also encouraged to send out a reminder email to staff approximately 1 month after the initial invitation was sent.

### Data Analysis

Quantitative data were analyzed using descriptive statistics of frequencies through Excel Microsoft 365, with continuous variables presented as means and SDs. Open-ended data were extracted verbatim and analyzed using an inductive qualitative approach, with transcripts coded into descriptive qualitative codes and then collapsed into themes using qualitative data analysis software (NVivo 12, QSR International). Initial coding was completed independently by 2 researchers (KJC and KM), and the development of themes was carried out collaboratively by 2 researchers (KJC and KM), with themes then validated by a third researcher (JC) to ensure rigor and enhance the trustworthiness of data analysis.

### Ethical Considerations

Consent was obtained from participants through the use of a question at the beginning of each survey. As survey responses were anonymous, participants could not withdraw their data after submission; however, participants were notified that they could withdraw consent by exiting the survey without submitting any responses. There was no compensation provided for participation. This study was approved by the Hamilton Integrated Research Ethics Board (REB #7691). That approval did not include the ability to publish verbatim survey responses.

## Results

### Quantitative

Between the period of June and August 2021, senior leaders of each survey group sent out survey requests to physicians and EMS paramedic staff, with response rates varying among each of the stakeholder groups sampled. Of the 10 ICs at the Hamilton General Hospital, 9 (90%) completed the survey. Of the 223 invited EM physicians, 51 (22.9%) completed the survey, and 93 (8.2%) of the 1138 invited EMS paramedics completed the survey.

Of the 51 EM physicians, 49 (96%) reported that they currently submit ECGs to ICs. Nearly all (50/51, 98%) did so through SMS text messages, while 39% (20/51) also reported sending ECGs via fax. All 9 ICs indicated that they received ECGs via SMS text message, while 33% (3/9) reported receiving ECGs also via fax. EMS paramedics reported that they rarely submitted prehospital ECGs to ICs (7/93, 8%), and physical ECGs were typically exchanged in person at patient handoff.

Among all groups, there was recognition that current practices for sharing ECGs allowed room for improvement, accepting that fax can be inconvenient and SMS text messages are not secure. When asked whether there was a need for a smartphone app to transmit ECGs, ICs (mean 6.67, SD 0.50), EM physicians (mean 5.57, SD 1.30), and EMS paramedics (mean 5.79, SD 1.45) consistently agreed one was needed. Furthermore, among ICs, EM physicians, and EMS paramedics, use of the app was believed to fit well into existing workflow processes, with mean Likert responses of 6.67 (SD 0.50), 5.65 (SD 1.31), and 5.56 (SD 1.77), respectively. Staff also noted feeling comfortable with using an app for ECG transmission and review, with mean Likert responses of 6.44 (SD 0.88), 5.51 (SD 1.92), and 5.22 (SD 2.05) reported. Table 1 highlights viewpoints on the implementation of a smartphone app to transmit ECGs from each group.

**Table 1.** Mean and SD scores from 7-point Likert scale responses on the implementation of a smartphone app to transmit ECGs^a^ in cases of suspected STEMI^b^.

	Interventional cardiologists, mean (SD)	EM^c^ physicians, mean (SD)	EMS^d^ paramedics, mean (SD)
There is a need for a smartphone app for the transmission and review of ECGs	6.67 (0.5)	5.57 (1.3)	5.79 (1.45)
The implementation of a smartphone app fits well within existing work processes in my setting	6.67 (0.5)	5.65 (1.31)	5.56 (1.77)
Using a smartphone app for synchronous review of ECGs will help to reduce the time to STEMI treatment	6 (1.32)	5 (1.55)	5.17 (1.65)
Using a smartphone app for synchronous review of ECGs will help to reduce the number of false STEMI activations	6.33 (1.12)	4.59 (1.66)	5.69 (1.22)
How comfortable are you with the idea of using a smartphone app for the transmission and review of ECGs?	6.44 (0.88)	5.51 (1.92)	5.22 (2.05)

a ECG: electrocardiogram.

b STEMI: ST-elevation myocardial infarction.

c EM: emergency medicine.

d EMS: emergency medical service.

### Qualitative

Through inductive qualitative analysis, 24 codes were identified for ICs, 46 codes were identified for EM physicians, and 57 codes were identified for EMS paramedics (Table 2). The codes were grouped into four key themes, which housed both barriers and enablers to app implementation: (1) technological issues and constraints; (2) perceived capabilities, alternatives, and comparisons of the app; (3) attitudes and beliefs toward the app; and (4) impact on current workflow and requirements for implementation (Table 3). Table 4 demonstrates how the identified barriers were mapped to implementation strategies.

Commonly reported barriers to implementation were concerns over technological failures within the app. Given that STEMI activation is a medical emergency, there were concerns that app updates could be required during urgent scenarios, making the app unusable and thus affecting clinical care. EMS paramedic providers and physicians practicing at hospitals with unreliable internet signals also expressed concerns about their ability to reliably call and transmit ECGs using their smartphones. Another important barrier was the uncertainty about the proven risks and benefits of the app, as this is a new technology without extensive real-world data. Privacy and security of the tool to transmit personal health information was raised. There was concern that the app could take more time than the current clinical workflows, thus worsening the process of STEMI activation. Respondents also expressed a desire to follow up with referring or accepting physicians to discuss the case in the future, which is a feature not offered via the app.

**Table 2.** Codebook created for the analysis of themes on the barriers and enablers to app implementation.

	Interventional cardiologists	EM^a^ physicians	EMS^b^ paramedics
Technological issues and constraints	App needs to work well App not working App requires redownloading Biometric authentication Log-in and password issues	App data security App not working App registration process Backup option needed in case app fails Cell signal issues Concerns over alignment of app with privacy standards Patient data privacy Reliability of app technology Speed of ECG^c^ transmission Technology failure User log-in and password issues Wi-Fi signal issues	App data security App not working Backup option needed in case app fails Cell signal issues Concerns over alignment of app with privacy standards No time to update app Patient data privacy Reliability of app technology Technology failure
Perceived capabilities, alternatives, and comparisons of the app	Alternative to app: critical hotline Comparison to current practices Desire to send or receive additional clinical information Follow-up capacity to referring physician Impact on patient care Storage for physician dialogue	Access to app by temporary physicians Comparison with current practice Designated phone in emergency department ECG image quality Follow-up capability to referring physician Impact on patient care In favor of app Relative advantage Storage for physician dialogue Text/fax along with dialogue considered safest Usability across multiple platforms	Impact on patient care Alternative to app – ECG monitor direct submission Alternative to app – iPad Alternative to app – cellular enabled computers Alternative to app – video telemedicine Alternative to app – email Benefit of IC^d^ consultation Comparison with current practice Confirmation needed that ECG sent successfully ECG image quality Limitations of point-in-time ECGs
Attitudes and beliefs toward the intervention	Ease of use Evaluate adoption in clinical practice Evaluate unexpected consequences Interventionalist requested texting Positive experience with app	Alternative to app: Epic Systems Corporation’s electronic medical records Alternative to app: hypercare App implementation creates opportunity for error Data security on personal device Ease of use Frequency of use Interventionalist requested texting Positive experience with app Resistance to new system process Responsibility – unit staff/coordinators Uncertainty about harms and benefits Use of personal cellular data Use of personal device	App not needed Appropriately shifts decision-making Comfort with using technology Concerns over liability if app does not work Ease of use Impact on paramedic ECG interpretation skills Impact on patient care In favor of app Negative response from ICs Not in favor of app Paramedics can diagnose well without the app Reduces paramedic autonomy Testing of app required before implementation Uncertainty about harms and benefits Use of personal cellular data Use of personal device Use of service provided smartphone
Impact on current workflow and requirements for implementation	Managing expectations of emergency medicine physicians More frequent review by ICs Need for contact information of referring physician Opportunity for physician dialogue Scope of appropriate patient cases Time to complete app process Time to reset password Timely access to ECG by IC	Administrative burden Asynchronous training Need for physician dialogue No time to update app Quick training for new physicians Response time by IC Scope of appropriate patient cases Time to complete app process Training recipients – unit coordinators Video training	Increased scene time More frequent review by ICs Need for physician dialogue Need for training Outcome – transport time Outcome – false activation Outcome – increase in-field time Outcome – cost Outcome – timely treatment Practicalities of using phone while in the ambulance Practicalities of using phone while in the field Practicalities of using phone while wearing PPE^e^ Public image of using phone on scene Response time by IC Time to complete app process Training – case scenario Training – ECG interpretation Training – in-person Training – phone and text support Training – video training

a EM: emergency medicine.

b EMS: emergency medical service.

c ECG: electrocardiogram.

d IC: interventional cardiologist.

e PPE: personal protective equipment.

**Table 3.** Barriers and enablers related to the implementation of a smartphone app to transmit ECGs^a^ in cases of suspected STEMI^b^ across all sampled groups.

Themes and barriers		Enabler	CFIR^c^ construct	CFIR construct definition [18]	ICs^d^	EM^e^ physicians	EMS^f^ paramedics
**Technological issues and constraints**							
	Technology failure	—^g^	Complexity	Perceived difficulty of implementation, reflected by duration, scope, radicalness, disruptiveness, centrality, and intricacy, and number of steps required to implement.	✓	✓	✓
Cell signal issues	—	Complexity	Perceived difficulty of implementation, reflected by duration, scope, radicalness, disruptiveness, centrality, and intricacy, and number of steps required to implement.	—	✓	✓
User log-in, password recall, and registration issues	—	Complexity	Perceived difficulty of implementation, reflected by duration, scope, radicalness, disruptiveness, centrality, and intricacy, and number of steps required to implement.	✓	✓	✓
—	Speed of ECG submission	Relative advantage	Stakeholders’ perception of the advantage of implementing the intervention versus an alternative solution.	✓	✓	✓
ECG image quality	—	Knowledge and beliefs about the intervention	Individuals’ attitudes toward and value placed on the intervention as well as familiarity with facts, truths, and principles related to the intervention.	—	✓	✓
—	ECG image quality	Knowledge and beliefs about the intervention	Individuals’ attitudes toward and value placed on the intervention as well as familiarity with facts, truths, and principles related to the intervention.	✓	—	—
Privacy and security	—	Knowledge and beliefs about the intervention	Individuals’ attitudes toward and value placed on the intervention as well as familiarity with facts, truths, and principles related to the intervention.	—	✓	✓
Use of personal data allowance	—	Knowledge and beliefs about the intervention	Individuals’ attitudes toward and value placed on the intervention as well as familiarity with facts, truths, and principles related to the intervention.	—	✓	—
**Perceived capabilities, alternatives, and comparisons of the app**							
	Time to complete app process	—	Complexity	Perceived difficulty of implementation, reflected by duration, scope, radicalness, disruptiveness, centrality, and intricacy, and number of steps required to implement.	✓	✓	✓
Desire to send/receive additional clinical information	—	Compatibility	The degree of tangible fit between meaning and values attached to the intervention by involved individuals, how those align with individuals’ own norms, values, and perceived risks and needs, and how the intervention fits with existing workflows and systems.	—	—	✓
—	Opportunity for physician dialogue to discuss borderline cases	Compatibility	The degree of tangible fit between meaning and values attached to the intervention by involved individuals, how those align with individuals’ own norms, values, and perceived risks and needs, and how the intervention fits with existing workflows and systems.	—	✓	✓
Opportunity for follow-up between referring physician and IC	—	Compatibility	The degree of tangible fit between meaning and values attached to the intervention by involved individuals, how those align with individuals’ own norms, values, and perceived risks and needs, and how the intervention fits with existing workflows and systems.	✓	✓	—
Alternative practices (fax) believed to align more with privacy standards	—	Relative advantage	Stakeholders’ perception of the advantage of implementing the intervention versus an alternative solution.	—	✓	✓
—	Secure solution compared to texting images	Relative advantage	Stakeholders’ perception of the advantage of implementing the intervention versus an alternative solution.	✓	✓	—
**Attitudes and beliefs toward the intervention**							
	—	Ease of use	Complexity	Perceived difficulty of implementation, reflected by duration, scope, radicalness, disruptiveness, centrality, and intricacy, and number of steps required to implement.	✓	—	—
—	In favor of app	Knowledge and beliefs about the intervention	Individuals’ attitudes toward and value placed on the intervention as well as familiarity with facts, truths, and principles related to the intervention.	✓	✓	✓
Resistance to new system procedure	—	Individual stage of change	Characterization of the phase an individual is in, as he or she progresses toward skilled, enthusiastic, and sustained use of the intervention.	—	✓	✓
—	Positive experience with using app	Individual stage of change	Characterization of the phase an individual is in, as he or she progresses toward skilled, enthusiastic, and sustained use of the intervention.	✓	—	—
Uncertainty about harms and benefits	—	Evidence, strength, and quality	Stakeholders’ perceptions of the quality and validity of evidence supporting the belief that the intervention will have desired outcomes.	—	✓	✓
Need for evaluation of app in clinical practice	—	Evidence, strength, and quality	Stakeholders’ perceptions of the quality and validity of evidence supporting the belief that the intervention will have desired outcomes.	—	✓	✓
—	Potentially improve patient outcomes	Relative advantage	Stakeholders’ perception of the advantage of implementing the intervention versus an alternative solution.	✓	✓	—
**Impact on current workflow and requirements for implementation**							
	Lead to more frequent review by ICs	—	Complexity	Perceived difficulty of implementation, reflected by duration, scope, radicalness, disruptiveness, centrality, and intricacy, and number of steps required to implement.	✓	—	—
Administrative burden	—	Complexity	Perceived difficulty of implementation, reflected by duration, scope, radicalness, disruptiveness, centrality, and intricacy, and number of steps required to implement.	—	✓	✓
Time needed to update app or reset password if needed	—	Complexity	Perceived difficulty of implementation, reflected by duration, scope, radicalness, disruptiveness, centrality, and intricacy, and number of steps required to implement.	✓	✓	✓
—	Response time by IC	Relative advantage	Stakeholders’ perception of the advantage of implementing the intervention versus an alternative solution.	—	✓	✓
—	Timely access to ECG by IC	Relative advantage	Stakeholders’ perception of the advantage of implementing the intervention versus an alternative solution.	✓	✓	✓
—	Diverse training options available	Engaging	Attracting and involving appropriate individuals in the implementation and use of the intervention through a combined strategy of social marketing, education, role modeling, training, and other similar activities.	✓	✓	✓
Increase on-scene time in the field	—	Compatibility	The degree of tangible fit between meaning and values attached to the intervention by involved individuals, how those align with individuals’ own norms, values, and perceived risks and needs, and how the intervention fits with existing workflows and systems.	—	—	✓
Degradation of ECG interpretation skills	—	Other personal Attributes	A broad construct to include other personal traits such as tolerance of ambiguity, intellectual ability, motivation, values, competence, capacity, and learning style.	—	—	✓

a ECG: electrocardiogram.

b STEMI: ST-elevation myocardial infarction.

c CFIR: Consolidated Framework for Implementation Research.

d IC: interventional cardiologist.

e EM: emergency medicine.

f EMS: emergency medical service.

g Not applicable.

## Discussion

### Principal Results

Responses from the preimplementation surveys addressed the objectives of this study, which sought to develop an understanding of the barriers and enablers to implementing an app that facilitates emergency cardiac communication. The primary use case is to securely transmit ECG images and enable telephone calls directly via the app in the case of suspected STEMI in a large health region in Ontario, Canada. Although previous studies have examined the diagnostic use of ECG image transmission via an app for STEMI care [11,22,23], this paper is the first to report on the barriers and enablers to implementing such an app from target users, allowing for tailored implementation strategies to be used in future to increase uptake. Overall, survey findings demonstrated agreement on the perceived need for an app and consensus that the app would fit well within existing workflow processes, reduce time to STEMI treatment, and reduce inappropriate activation of the on-call IC team. Commonly reported barriers were concerns over technological challenges, privacy issues, and cell phone reception strength.

While all 3 groups shared concerns over potential technological challenges, privacy issues, and cell phone reception strength, there were differences in the barriers and enablers reported by each group (Table 3). As the app was previously implemented in the IC group during the pilot phase in 2020, several of the barriers expressed by the EM physicians and EMS paramedics were not reported by the ICs. For example, the ICs were aware of app functionality and ECG image quality and so did not report these as a barrier to use. However, the majority of EM physicians and all EMS paramedics had no prior experience with using the app at the time of survey collection, and so responses illustrate apprehension of the unknown. Across each stakeholder group, common enablers were the expected speed of ECG image sharing and the associated timely access to that knowledge distribution. All groups reported comments that were both in favor and against app implementation. Given the rapid growth of smartphone technology in recent decades and its increasing adoption among consumers, it is unsurprising that survey responses reflected a high level of comfort with using an app to transmit ECG images. Furthermore, mobile health technology has the potential to enhance system efficiencies and streamline workflow processes, which may account for our findings indicating both a recognized need for an app to transmit ECG images and its perceived compatibility with existing practices. From the barriers and enablers that were identified in the survey responses, select CFIR constructs were found to be represented more frequently than others (Table 3). Under the technological issues and constraints theme, CFIR constructs such as complexity and knowledge and beliefs about the intervention were commonly referenced. This indicated that the individual’s personal perception on how difficult the app would be to use and their personal attitudes toward using the app for cases of suspected STEMI were going to be contributing factors to whether they took the necessary steps to download and use the app. In the perceived capabilities and comparisons theme, CFIR constructs of compatibility and relative advantage were frequently cited. Participants’ willingness to use the app depended on whether they saw it as valuable, fulfilling a clinical need, and offering advantages over existing ECG transmission methods. In the attitudes and beliefs toward the intervention theme, there were no dominant CFIR constructs; however, responses indicated that participant perception on the evidence supporting the need for an app to transmit ECG images influenced whether they themselves would download and use it. Finally, in the impact on current workflow and requirements for implementation theme, complexity and relative advantage were again most commonly referenced, demonstrating that the perceived difficulty of incorporating the app into their current workflow processes and their perception on the benefits to using the app, would impact uptake. Our findings of the barriers identified in the survey responses, combined with our analysis of the most commonly cited CFIR constructs, facilitated the development of implementation strategies for app use and scale-up.

Results from the web-based surveys allowed for the application of stakeholder-specific implementation science interventions that supported the uptake of the app on a wider scale across the health region. For example, we obtained an understanding of the preferred mechanism of training on app use and applied appropriate education strategies, such as offering Zoom drop-in question and answer training sessions at accessible times during the day, to accommodate shift work hours. Furthermore, we cultivated relationships with opinion leaders (EM chiefs) and early adopters and sought advice from experts in implementation science research to maximize survey responses and increase uptake and use of the app. To address password recall concerns among ICs, biometric authentication was introduced to streamline log-in. Finally, key barriers related to concerns over technological challenges and privacy issues were addressed with the development and distribution of educational materials, such as user guides that highlighted multiple successful security tests from internal privacy, security, and information technology staff along with external third-party vendors. Table 4 illustrates the barriers by theme and the implementation strategies that were used to facilitate the uptake and use of the app.

This study focuses on a specific region in Ontario, Canada; however, the results can be generalized to other regions or countries where an app is being considered for inclusion in patient care by health care providers, as noted by the similarity of findings to published literature. A systematic review of barriers and facilitators to using digital health technologies representing North America, Europe, Asia, Africa, and Latin America highlighted infrastructure and technical barriers as well as privacy and security as commonly reported concerns by health care providers when using app-based technology [24]. As reported in our findings, concerns over insufficient network and connectivity speed were found, with a higher prevalence of these barriers being reported by those working in rural or remote regions. Furthermore, a published study on app use among nurses reported similar findings, highlighting perceived usefulness and ease of use as key factors influencing adoption [25]. The alignment of this study’s findings with previously published research on barriers and enablers to app use by health care providers underscores the importance of engaging users before implementation, which can assist in developing targeted strategies to enhance the adoption and integration of new technology.

These findings are relevant to clinical practice improvements as they highlight the potential for mobile health technology to enhance communication in STEMI care and improve coordination among health care providers. By addressing identified barriers and leveraging enablers, the successful integration of an app for ECG transmission could improve the efficiency of STEMI activation, reduce unnecessary patient transfers, and ultimately enhance patient outcomes by minimizing delays in critical care. While emerging technology can allow for the integration of a machine learning model to enhance the ECG diagnosis of acute coronary occlusion, research testing the precision and sensitivity of such models combined the judgment of trained health professionals for ECG review [26]. The findings presented in this paper can support the integration of new technologies, including artificial intelligence–enabled apps, into acute STEMI care, as without understanding how to effectively implement and maintain an app in the acute management of STEMI, similar barriers are likely to hinder the successful adoption of artificial intelligence technologies in this setting.

Following this analysis of barriers and enablers to implementation, further research is needed to evaluate the impact of mobile health technology on the care of suspected STEMI cases. Specifically, it is important to assess whether addressing the identified barriers and leveraging enablers leads to successful implementation strategies that enhance app adoption and sustained use. Further analysis from this implementation will explore data on app uptake, usage, and effectiveness, with findings to be published in subsequent papers.

**Table 4.** Mapping barriers identified from survey responses to implementation strategies.

Theme	Barrier	Implementation strategy
Technological issues and constraints	Potential to forget log-in credentials which could delay care Privacy and security Time needed to update app Technology failure	Biometric authentication (face/fingerprint ID) was enabled for ICs^a^ to assist with faster log-in and to address concerns over password recollection Targeted education messages were developed, which noted third-party security testing to relay privacy concerns App updates automatically In the app invitation email, users are encouraged to notify the study team should they have any issues with the app functionality or logging-in to the app
Perceived capabilities, alternatives, and comparisons of the app	Follow-up capacity to referring physician Usability across multiple platforms	Referring physician required to call the STEMI^b^ activation number through the app and speak to on-call IC, allowing for the opportunity to discuss patient care User guide and app invitation email provide links to both the Apple App Store and the Google Play Store
Attitudes and beliefs toward the app	Ease of use Resistance to new system process App believed to be unnecessary	User guide contains images depicting app simplicity Cultivated relationships with opinion leaders (EM^c^ chiefs) and early adopters from the pilot study With the support of EM chiefs, a newsletter was sent out to EM physicians that included important reminders about app use, a link to the education session recording, and a general update on app downloads across the region to demonstrate uptake by peers
Impact on current workflow and requirements for implementation	Concerns about how app would fit into existing workflows Asynchronous training	Developed a user guide with clearly structured guidance and step-by-step instructions on how and when the app should be used Hosted a collaborative web-based education session for EM physicians discussing the regional STEMI program and the app. A recording of the session was made available for asynchronous learning on a dedicated web page that also housed additional educational materials. In addition, scheduled web-based drop-in Q&A sessions at multiple time periods throughout the day during time of app launch, to accommodate shift work hours

a IC: interventional cardiologist.

b STEMI: ST-elevation myocardial infarction.

c EM: emergency medicine.

### Limitations

As the surveys were distributed prior to the majority of EM physicians and EMS paramedics gaining access to the app, there was limited understanding of app features, capabilities, and usability among these 2 groups. This made it difficult for participants to answer certain questions and was reflected in survey responses that noted inadequate knowledge affected their ability to reliably answer some questions. Nonetheless, this provided valuable, unbiased responses to questions aimed at understanding the need for such a tool. Additionally, as links to the survey were shared directly by senior leaders of each stakeholder group, it was challenging for the research team to manage follow-up emails that may have improved survey completion rates, as well as calculate accurate response rates. However, this was the preferred means of communication by the senior leaders to maintain anonymity, confidentiality, and avoiding coercion to respond to questionnaires. It is also possible that response rates were improved as participants were being directly asked to complete the survey by the chief or chair of their department. This study leveraged a convenience sampling strategy, which can introduce selection and sampling bias. As such, caution is necessary in the generalization of the results. Reassuringly, there was significant representation from each hospital and paramedic region with similar themes and responses for both the qualitative and quantitative questions.

### Conclusions

The implementation of system-level interventions in health care can be affected by unexpected barriers, leading to poor uptake and acceptance. In order to circumvent the challenges of implementing change to an organization’s culture, understanding the variables that influence or have an impact on the population’s willingness to accept and adopt the change is critical. This study exemplifies the importance of identifying barriers faced by the target population and using that knowledge to create successful implementation strategies to optimize research interventions. Results from the preimplementation surveys disseminated to ICs, EM physicians, and EMS paramedics helped inform the strategies for the SmartAMI-ACS app implementation program in Hamilton and the surrounding region. They will be vital in informing active scaling of the app into additional areas throughout the province and local ambulance services.

## Supplementary material

10.2196/60173 Multimedia Appendix 1 STEMI activation questionnaire: interventional cardiologists. STEMI: ST-elevation myocardial infarction.

10.2196/60173 Multimedia Appendix 2 STEMI activation questionnaire: emergency medicine physicians. STEMI: ST-elevation myocardial infarction.

10.2196/60173 Multimedia Appendix 3 STEMI activation questionnaire: paramedics. STEMI: ST-elevation myocardial infarction.

## Abbreviations

CFIR: Consolidated Framework for Implementation Research
ECG: electrocardiogram
EM: emergency medicine
EMS: emergency medical service
IC: interventional cardiologist
PCI: percutaneous coronary intervention
REDCap: Research Electronic Data Capture
SmartAMI: Strategic Management of Acute Reperfusion and Therapies in Acute Myocardial Infarction
STEMI: ST-elevation myocardial infarction
