# Supplementary material for: Exploring the Barriers and Facilitators to Implementing a Smartphone App for Physicians to Improve the Management of Acute Myocardial Infarctions: Multicenter, Mixed Methods, Observational Study
Source: JMIR Mhealth Uhealth. 2025 Jul 8;13:e60173. doi: 10.2196/60173 (PMC12262926; doi:10.2196/60173)
Supplement: Multimedia Appendix 1 [file mhealth-v13-e60173-s001.docx]

# STEMI Activation Questionnaire – **Interventional Cardiologists**

**ELECTRONIC SURVEY FORM**

## Introduction

You are being asked to fill out this brief survey to help us understand the interventional cardiologist's impression of how the STEMI paging system is currently being used, as well as your feedback on the use of a smartphone application for the transmission and review of ECGs. **The survey should take less than 5 minutes to complete.**

Please answer the following questions to the best of your ability. Your responses will be confidential; we do not collect identifying information. As such, once submitted, no data will be able to link this survey to your individual identity. You may skip questions that you are unable to answer. **Your participation with this survey is voluntary. Your consent is inferred when you complete and submit the form.**

Acronyms used in this survey:

- DNR: Do Not Resuscitate
- ECG: Electrocardiogram
- HIU: Heart Investigation Unit
- PCI: Percutaneous Coronary Intervention
- STEMI: ST Elevation Myocardial Infarction

## Patient Factors

Please rate your level of agreement with the following statement. (7-point Likert scale; strongly disagree =1 – strongly agree =7):

- Over the last five years, when I am on interventional cardiology call, I have noticed an increase in the number of STEMI pages from the HIU Hotline that are not accepted by myself for primary PCI.
  - [If 5 or above] Please select how often you encounter the following clinical scenarios for declining primary PCI. (7-point Likert scale; never– always):
    - Patient requires admission to ward or critical care but does not meet STEMI criteria.
    - Out of hospital cardiac arrest patient who does not meet STEMI criteria.
    - Goals of care do not include primary PCI, such as someone whose code status is DNR.

Do you have any comments about declining primary PCI? (open text box)

## Knowledge Factors

Please rate your level of agreement with the following statements. (7-point Likert scale; strongly disagree – strongly agree):

- In general, the knowledge base on STEMI diagnosis and management by referring health care providers has decreased over time.
- It would be helpful to have a feedback mechanism for health care providers who call in to the HIU Hotline.
- I would be willing to participate in a feedback system (such as sending a text with patient outcomes) for health care providers who call in to the HIU Hotline.

Do you have any comments about providers' STEMI knowledge and/or feedback mechanisms for providers who call in to the HIU Hotline? (open text box)

## Communication and System Factors

Please select how often the HIU Hotline is used for the following. (7-point Likert scale; never – always):

- To get an opinion from a cardiologist without there being clear STEMI criteria.
- To get information about system processes or to arrange for a non-urgent PCI.

Do you have any comments about how the hotline is currently being used? (open text box)

How do you currently receive ECGs from emergency medicine physicians/paramedics? Select all that apply.

- Fax
- Text Message
- Email
- Smartphone application

Please describe any other ways in which you receive ECGs for review. (open text box)

Please rate your level of agreement with the following statements. (7-point Likert scale; strongly disagree – strongly agree):

- There is a need for a smartphone application for the transmission and review of ECGs.
  - [If 3 or less] Please explain why you generally disagree with the statement: There is a need for a smartphone application for the transmission and review of ECGs. (open text box)
- The implementation of a smartphone application fits well within existing work processes in my setting.

Do you have any comments regarding your answers to the previous statements? (open text box)

Are there any complications or issues that may arise because of the implementation of a smartphone application for the transmission and review of ECGs? (Yes/No/Uncertain)

- [If Yes] Please explain why you selected "Yes". (open text box)
- [If Uncertain] Please explain why you selected “Uncertain”. (open text box)

How comfortable are you with the idea of using a smartphone application for the transmission and review of ECGs? (7-point Likert-scale; very uncomfortable – very comfortable)

Do you have any comments about your comfort level? (open text box)

Do you have any concerns about patient privacy regarding the use of a privacy-compliant smartphone application? (Y/N)

- [If Yes] What concerns do you have about patient privacy? (open text box)

Do you have any concerns about technical problems occurring with the smartphone application? (Y/N)

- [If Yes] What technical problems are you concerned about? (open text box)

What is your preferred type of training to support the use of a new smartphone application? Select all that apply.

- A scheduled, structured online training session
- Distribution of a summary document on how to use the app (with step-by-step instructions)
- Identification of, and access to, a key contact person who can answer questions about the app if necessary
- No training is needed

Please describe any other types of training that you would like to have available. (open text box)

Please rate your level of agreement with the following statements. (7-point Likert scale; strongly disagree – strongly agree):

- Using a smartphone application for synchronous review of ECGs will help to reduce time to STEMI treatment.
- Using a smartphone application for synchronous review of ECGs will help to reduce the number of false STEMI activations.

Do you have any comments about the possible implications of using a smartphone application for synchronous review of ECGs? (open text box)

Please include any additional comments or concerns about the smartphone application and/or its implementation. (open text box)
