# Supplementary material for: Exploring the Barriers and Facilitators to Implementing a Smartphone App for Physicians to Improve the Management of Acute Myocardial Infarctions: Multicenter, Mixed Methods, Observational Study
Source: JMIR Mhealth Uhealth. 2025 Jul 8;13:e60173. doi: 10.2196/60173 (PMC12262926; doi:10.2196/60173)
Supplement: Multimedia Appendix 3 [file mhealth-v13-e60173-s003.docx]

# STEMI Activation Questionnaire – **Paramedics**

**ELECTRONIC SURVEY FORM**

## Introduction

You are being asked to fill out this brief survey to help us understand the paramedic’s impression of how the STEMI paging system is currently being used, as well as your feedback on the use of a smartphone application for the transmission and review of ECGs. **The survey should take less than 5 minutes to complete.**

Please answer the following questions to the best of your ability. Your responses will be confidential; we do not collect identifying information. As such, once submitted, no data will be able to link this survey to your individual identity. You may skip questions that you are unable to answer. **Your participation with this survey is voluntary. Your consent is inferred when you complete and submit the form.**

Acronyms used in this survey:

- ECG: Electrocardiogram
- HIU: Heart Investigation Unit
- PCI: Percutaneous Coronary Intervention
- ROSC: Return of Spontaneous Circulation
- STEMI: ST Elevation Myocardial Infarction

## Location of Practice

Please indicate your primary location of practice.

- Brant Brantford Paramedic Service
- Haldimand County Paramedic Service
- Halton Region Paramedic Services
- Hamilton Paramedic Service
- Niagara Emergency Medical Services
- Norfolk County Paramedic Service
- Six Nations Paramedic Service
- Other
  - [If Other] You selected “Other”. Please type in your primary location. (open text box)

## Frequency of Use

Within the last year, how often have you used the HIU Hotline?

- Never
- 1-3 times
- 4-6 times
- 7 or more times

## Patient Factors

How often have you used the HIU Hotline for the following patient profiles? (Never, Sometimes, Always).

- Patients who have had out of hospital cardiac arrest followed by ROSC without ST elevations on ECG.
  - [If Sometimes or Always] Please elaborate (with some clinical scenarios) on your response that you “sometimes” or “always” use the hotline for patients who have out of hospital cardiac arrest followed by ROSC without ST elevations on ECG. (open text box)
- Patients who are having a STEMI, but their goals of care do not include coronary angiography.
  - [If Sometimes or Always] Please elaborate (with some clinical scenarios) on your response that you “sometimes” or “always” use the hotline for patients who are having a STEMI, but their goals or care do not include coronary angiography. (open text box)
- Patients who are not a clear STEMI, to clarify diagnostic uncertainty.
  - [If Sometimes or Always] Please elaborate (with some clinical scenarios) on your response that you “sometimes” or “always” use the hotline for patients who are not a clear STEMI, to clarify diagnostic uncertainty. (open text box)

Do you have any other comments about HIU Hotline use? (open text box)

## Knowledge Factors

Do you know the indications for activating the STEMI Hospital Bypass Protocol through the HIU Hotline? (Y/N)

How often do you contact the HIU Hotline if the field ECG reads, “Acute MI and/or ST-elevation” regardless of patient symptoms or presentation? (7-point Likert scale; never - always)

How often do you use the HIU Hotline to help guide medical management of a STEMI? (7-point Likert scale; never - always)

Do you have any comments regarding your answers to the previous questions? (open text box)

Please rate your level of agreement with the following statements. (7-point Likert scale; strongly disagree – strongly agree):

- I feel comfortable with diagnosing and managing a STEMI while transporting a patient to the cardiac catheterization lab.
- I would like to have access to regional STEMI resources and management, including STEMI activation and management guidelines.
- I would find it helpful to have a feedback mechanism for patient outcomes where I could look at a patient’s final diagnosis to determine if they were appropriately referred to the cardiac catheterization lab.
  - [If 5 or above] Please specify your preferred mechanism of feedback (e.g., text message, phone call, phone app, manual lookup through patient chart, etc.). (open text box)

Do you have any comments regarding your answers to the previous statements? (open text box)

## Communication and System Factors

Please rate your level of agreement with the following statement. (7-point Likert scale; strongly disagree – strongly agree):

- I have appropriate educational resources when managing unwell cardiac patients in my clinical practice setting.

Do you have any comments about educational resources, related to the management of unwell cardiac patients? (open text box)

Do you currently submit ECGs to interventional cardiologists? (Y/N)

- [If Yes] How do you currently submit ECGs to interventional cardiologists? Select all that apply.
- Fax
- Text Message
- Email
- [If Yes] Please describe any other methods/formats that you use to submit ECGs. (open text box)

Please rate your level of agreement with the following statements. (7-point Likert scale; strongly disagree – strongly agree):

- There is a need for a smartphone application for the transmission and review of ECGs.
  - [If 3 or less] Please explain why you generally disagree with the statement: There is a need for a smartphone application for the transmission and review of ECGs. (open text box)
- The implementation of a smartphone application fits well within existing work processes in my setting.

Do you have any comments regarding your answers to the previous statements? (open text box)

Are there any complications or issues that may arise because of the implementation of a smartphone application for the transmission and review of ECGs? (Yes/No/Uncertain)

- [If Yes] Please explain why you selected "Yes". (open text box)
- [If Uncertain] Please explain why you selected “Uncertain”. (open text box)

How comfortable are you with the idea of using a smartphone application for the transmission and review of ECGs? (7-point Likert-scale; very uncomfortable – very comfortable)

Do you have any comments about your comfort level? (open text box)

Do you have any concerns about patient privacy regarding the use of a privacy-compliant smartphone application? (Y/N)

- [If Yes] What concerns do you have about patient privacy? (open text box)

Do you have any concerns about technical problems occurring with the smartphone application? (Y/N)

- [If Yes] What technical problems are you concerned about? (open text box)

What is your preferred type of training to support the use of a new smartphone application? Select all that apply.

- A scheduled, structured online training session
- Distribution of a summary document on how to use the app (with step-by-step instructions)
- Identification of, and access to, a key contact person who can answer questions about the app if necessary
- No training is needed

Please describe any other types of training that you would like to have available. (open text box)

Please rate your level of agreement with the following statements. (7-point Likert scale; strongly disagree – strongly agree):

- Using a smartphone application for synchronous review of ECGs will help to reduce time to STEMI treatment.
- Using a smartphone application for synchronous review of ECGs will help to reduce the number of false STEMI activations.

Do you have any comments about the possible implications of using a smartphone application for synchronous review of ECGs? (open text box)

Please include any additional comments or concerns about the smartphone application and/or its implementation. (open text box)
